# Identifying Dendritic Cell–Related Genes Through a Co-Expression Network to Construct a 12-Gene Risk-Scoring Model for Predicting Hepatocellular Carcinoma Prognosis

**DOI:** 10.3389/fmolb.2021.636991

**Published:** 2021-05-24

**Authors:** Chaoyuan Huang, Xiaotao Jiang, Yuancheng Huang, Lina Zhao, Peiwu Li, Fengbin Liu

**Affiliations:** ^1^ The First Clinical Medical School, Guangzhou University of Chinese Medicine, Guangzhou, China; ^2^ Department of Gastroenterology, The First Affiliated Hospital of Guangzhou University of Chinese Medicine, Guangzhou, China

**Keywords:** hepatocellular carcinoma, immune-related gene, overall survival, risk-scoring model, co-expression network construction

## Abstract

The prognostic prediction of hepatocellular carcinoma (HCC) is still challenging. Immune cells play a crucial role in tumor initiation, progression, and drug resistance. However, prognostic value of immune-related genes in HCC remains to be further clarified. In this study, the mRNA expression profiles and corresponding clinical information of HCC patients were downloaded from public databases. Then, we estimated the abundance of immune cells and identified the differentially infiltrated and prognostic immune cells. The weighted gene co-expression network analysis (WGCNA) was performed to identify immune-related genes in TCGA cohort and GEO cohort. The least absolute shrinkage and selection operator (LASSO) Cox regression model was applied to establish a risk-scoring model in the TCGA cohort. HCC patients from the GSE14520 datasets were utilized for risk model validation. Our results found that high level of dendritic cell (DC) infiltration was associated with poor prognosis. Over half of the DC-related genes (58.2%) were robustly differentially expressed between HCC and normal specimens in the TCGA cohort. 17 differentially expressed genes (DEGs) were found to be significantly associated with overall survival (OS) by univariate Cox regression analysis. A 12-gene risk-scoring model was established to evaluate the prognosis of HCC. The high-risk group exhibits significantly lower OS rate of HCC patients than the low-risk group. The risk-scoring model shows benign predictive capacity in both GEO dataset and TCGA dataset. The 12-gene risk-scoring model may independently perform prognostic value for HCC patients. Receiver operating characteristic (ROC) curve analysis of the risk-scoring model in GEO cohort and TCGA cohort performed well in predicting OS. Taken together, the 12-gene risk-scoring model could provide prognostic and potentially predictive information for HCC. SDC3, NCF2, BTN3A3, and WARS were noticed as a novel prognostic factor for HCC.

## Introduction

Liver cancer is the fourth most commonly diagnosed cancers and sixth in terms of leading cause among the cancer-related deaths in the world (Villanueva, 2019). Hepatocellular carcinoma (HCC), accounting for three-quarters of liver cancer, is considered to be the most prevalent histological type of primary liver cancer (Kim et al., 2016). HCC is attributed to multiple etiologies, including chronic hepatitis virus infection and alcoholic or nonalcoholic fatty liver disease (Yang et al., 2019). Recently, studies found that the tumor microenvironment (TME) is tightly involved with tumor development and progression (Chu and et al., 2019). TME serves a pivotal role in HCC progression, recurrence, and metastasis. The HCC microenvironment includes various cells; among all, immune cells are of paramount importance to not only tumor initiation and progression but also drug resistance (Zhou et al., 2016). The immune cells and their secretory substances may create an environment that exacerbates tumor progression (Zhou et al., 2019).

High-level heterogeneity of HCC adds to the difficulty in predicting prognosis of HCC (Huang et al., 2019). Immune-related parameters have been reported to predict the prognosis of patients with HCC, elucidating that the significance of immune status for determining the outcomes of HCC (Long et al., 2019). The presence of CD8+ Cytotoxic T lymphocytes (CTLs) in HCC tissue is beneficial for better survival situation (Fu et al., 2019). CSF1R expression in macrophages exerts an essential role in the interaction between macrophages and HCC cells (Chen S. et al., 2019). The molecular mechanisms underlying the interaction between hepatoma cells and macrophages may provide a novel vision for the therapeutic strategies of HCC (Tian et al., 2020).

The least absolute shrinkage and selection operator (LASSO) Cox regression analysis was first proposed in 1997 by Tibshirani, and simulations indicated that the LASSO could be more accurate than stepwise selection since LASSO reduces the estimation variance while providing an interpretable final model (Tibshirani, 1997). Except that this prognostic model has a long history, LASSO Cox regression has been widely applied to construct a prognostic model in multiple researches (Zong et al., 2010; Liang et al., 2019; Liu et al., 2019; Wu et al., 2019; Yue et al., 2019; Li et al., 2020; Liang et al., 2020). ImmuCellAI (Immune Cell Abundance Identifier) is a tool to estimate the abundance of 24 immune cells from gene expression dataset, including RNA-Seq and microarray data, and ImmuCellAI result-based model in tumor immune infiltration estimation demonstrates high accuracy and unique function (Miao et al., 2020).

Based on the fact that immune cells have significant value in evaluating the prognosis of various cancers, especially HCC (Zhuang et al., 2020), this study constructs the prognosis model of HCC through identifying immune-related genes co-expressed with immune cells which were associated with the prognosis of HCC. In this study, Immune Cell Abundance Identifier (ImmuCellAI), weighted gene co-expression network analysis (WGCNA), LASSO Cox analysis, receiver operating characteristic (ROC) curve analysis, univariate Cox analysis, and multivariate Cox analysis were applied to identify immune-related genes in the HCC microenvironment and construct a risk-scoring model, which exhibited benign prognostic value in TCGA cohort and GEO cohort. Univariate and multivariate Cox analyses showed our risk-scoring model was the independent prognostic factor for overall survival (OS) in both cohorts. In summary, our risk-scoring model can precisely predict OS for patients with HCC.

## Materials and Methods

**FIGURE 1:**
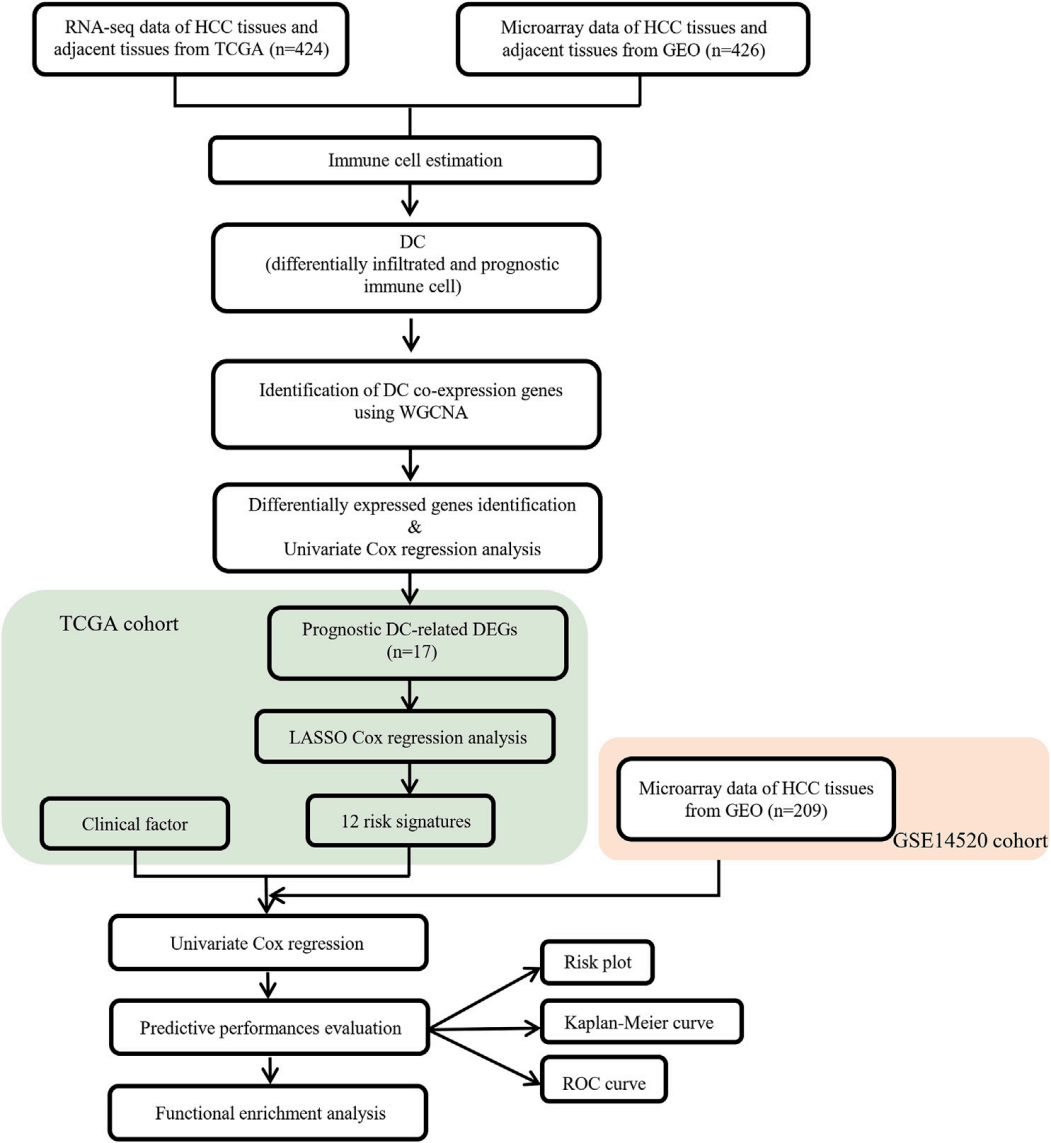
Flow chart of data collection and analysis.

The flow chart of this study is shown in Figure 1.

### Data Collection and Preprocessing

The mRNA expression profiles and corresponding clinical data of liver cancer samples in The Cancer Genome Atlas (TCGA) were retrieved from the University of California Santa Cruz Xena (https://xenabrowser.net/datapages/). 424 samples with mRNA expression and clinical data were obtained, including 374 HCC samples and 50 adjacent normal samples. Gene expression data and corresponding clinical information of GSE14520 datasets, totally including 209 patients, were downloaded from the Gene Expression Omnibus (GEO) database. Characteristics of included datasets are shown in Table 1.

**TABLE 1:** Clinical characteristics of the HCC patients used in this study.

	TCGA cohort	GEO cohort
Number of patients	371	209
Age (median, range)	61 (16–90)	51 (21–77)
**Gender(%)**		
Female	120 (32.3%)	26 (12.4%)
Male	251 (67.7%)	183 (87.6%)
**Race**		
White	185 (49.9%)	NA
Asian	158 (42.6%)	NA
Black or American	18 (4.8%)	NA
Unknown	10 (2.7%)	NA
**Grade(%)**		
Grade 1	55 (14.8%)	NA
Grade 2	178 (50.0%)	NA
Grade 3	120 (32.3%)	NA
Grade 4	13 (3.5%)	NA
Unknown	5 (1.3%)	NA
**Stage (%)**		
Ⅰ	174 (46.9%)	90 (43.1%)
Ⅱ	85 (22.9%)	74 (35.4%)
Ⅲ	84 (22.6%)	43 (20.6%)
Ⅳ	4 (1.1%)	NA
unknown	24 (6.4%)	2 (0.9%)
**Child–Pugh(%)**		
A	222 (59.8%)	NA
B	21 (5.7%)	NA
C	1 (0.3%)	NA
unknown	127 (34.2%)	NA
**Main tumor Size(%)**		
>5 cm	NA	75 (35.9%)
≤5 cm	NA	133 (63.6%)
Unknown	NA	1 (0.5%)
**Survival status**		
OS days (median)	602	1,581

### Estimation of Immune Cell Abundance

ImmuCellAI (http://bioinfo.life.hust.edu.cn/web/ImmuCellAI/) is a powerful and unique method for accurately estimating the tumor immune infiltration of 24 immune cell types, especially T-cell properties. Therefore, gene expression profiles of GSE14520 and HCC TCGA cohorts were uploaded to ImmuCellAI to estimate the abundance of immune cells.

### Identification of Differentially Infiltrated and Prognostic Immune Cells

The differentially infiltrated immune cells between tumor tissues and adjacent tissues were identified using the “limma” R package in the GSE14520 and HCC TCGA cohorts with a *p*-value <0.05. A Kaplan–Meier analysis was conducted to identify immune cells significantly associated with overall survival (OS). The patients would be grouped into high expression and low expression according to the median expression of each immune cell while conducting the Kaplan–Meier analysis. Overlapping immune cells with differential infiltration and prognostic value in the GSE14520 and HCC TCGA cohorts were considered as the hub immune cells and subjected to construct a related prognostic model.

### Construction of Gene Co-expression Network

Top 25% genes with the largest variance differences were applied to construct weight gene co-expression networks in GSE14520 and HCC TCGA cohort, respectively, via utilizing the “WGCNA” package in R software. The value of soft threshold power was confirmed at the point of the scale-free topology—R^2 exceeding 0.85. Genes with similar expression patterns were distributed to modules *via* average linkage hierarchical clustering under the circumstances of the minimum size of module, which was set to 30.

### Identification of Hub Modules

The correlations between the module eigengenes (MEs) and the differential infiltration levels of immune cells were calculated by Pearson’s correlation test in GSE14520 and HCC TCGA cohort, respectively, in order to identify the module paramountly correlating with the hub immune cell infiltration. *p*-value <0.05 was set as the cutoff value. Then, intersection of the modules with consistent correlation direction between GSE14520 and HCC TCGA cohorts was applied, and the overlapping genes were run by KEGG pathway enrichment analysis for identifying their potential functions and hub modules. The cutoff criterion was adjusted *p*-value <0.05. The overlapping genes were considered as hub genes associated with the hub immune cell infiltration.

### Identification of Differentially Expressed and Prognostic Genes

The differentially expressed hub genes between tumor specimens and adjacent specimens were identified using the “limma” R package in the primary cohort with a false discovery rate (FDR) <0.05 in the TCGA primary cohort. Univariate Cox analysis was conducted to identify genes closely associated with OS. The protein–protein interaction network (PPI) for the overlapping prognostic differentially expressed genes (DEGs) was performed by the STRING database (version 11.0). Overlapping genes with the characteristics of differential expression and prognostic value in the TCGA cohort were extracted to construct a prognostic model.

### Construction and Validation of the Prognostic Immune Cell–Related Risk-Scoring Model

Based on the expression of overlapping genes with differential expression and prognostic value as well as survival data, the “glmnet” R package was applied for the LASSO Cox regression analysis to further select and shrink predictors. The optimal value of penalty parameter (λ) was determined according to 10 cross-validations. Risk score of each patient was calculated based on the following formula: risk score = e^sum(each gene’s expression × corresponding coefficient)^. The median value of the risk score was considered as the cutoff value that categorized the patients into high-risk and low-risk groups. To explore the distribution of different groups, principal component analysis (PCA) and t-distributed stochastic neighbor embedding (t-SNE) were conducted using the “stats” and “Rtsne” R package, respectively. Survival rate between two groups was compared using a Kaplan–Meier survival curve. To evaluate the model’s predictive ability, a time-dependent ROC curve analysis was conducted using “survival ROC” package.

### Functional Enrichment Analysis

To explore the biological function associated with the risk, the enrichment analysis of Gene Ontology (GO) and Kyoto Encyclopedia of Genes and Genomes (KEGG) was carried out on the basis of the DEGs between high-risk and low-risk groups, by using the “cluster Profiler” R package (Jiang and et al., 2021). The Benjamini–Hochberg (BH) method was used to adjust the *p* values.

## Results

### The Immune Cell Abundance Estimation and Pivotal Immune Cell Identification

**FIGURE 2:**
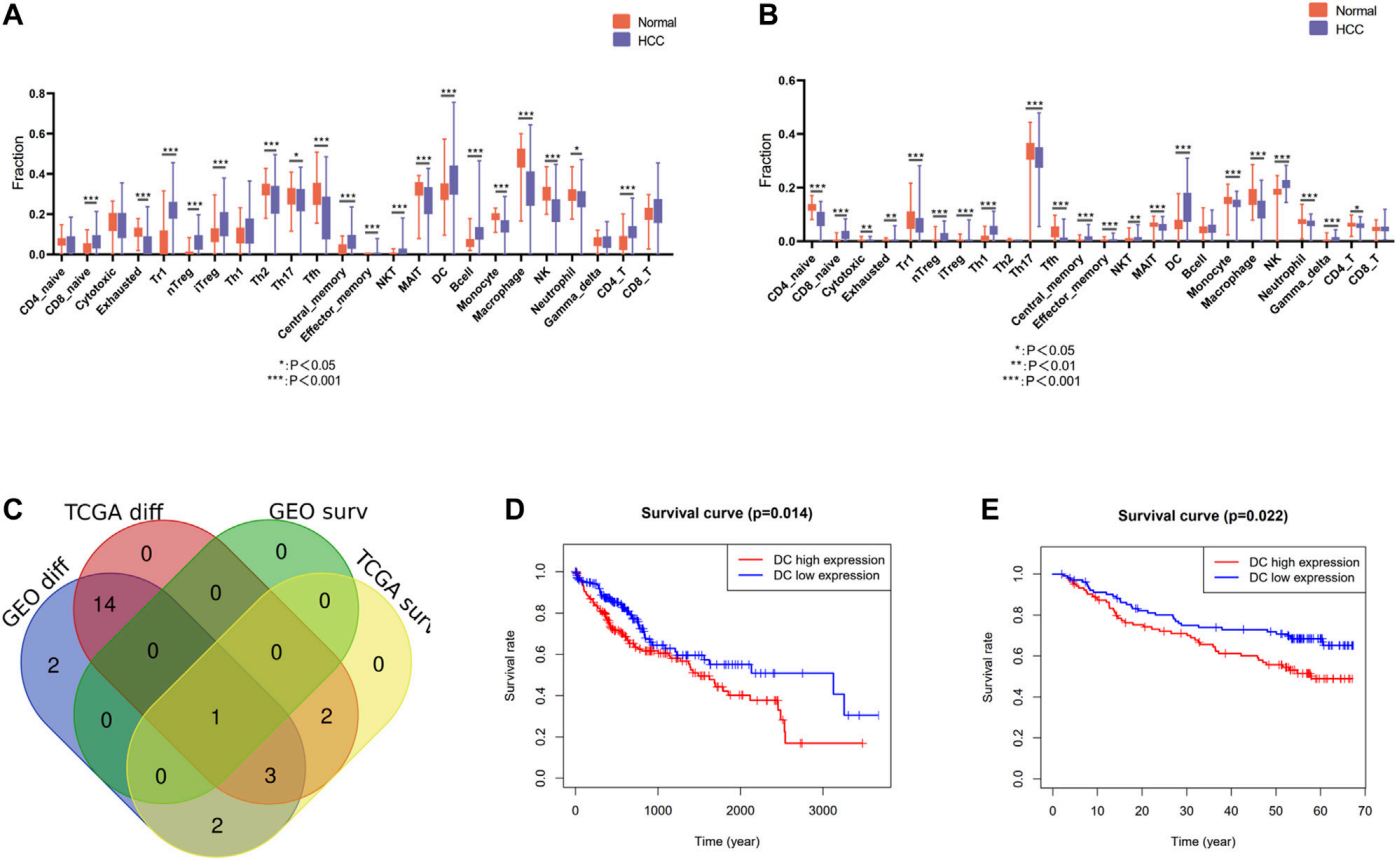
Identification of differentially infiltrated and prognostic immune cells. **(A)** Differentially infiltrated immune cells between HCC tissues and nontumor tissues in the TCGA cohort. **(B)** Differentially infiltrated immune cells between HCC tissues and nontumor tissues in the GEO cohort. **(C)** Venn diagram of differentially infiltrated and prognostic immune cells in TCGA cohort and GEO cohort. **(D)** Kaplan–Meier analysis of DCs in the TCGA cohort **(E)** Kaplan–Meier analysis of DCs in the GEO cohort.

The infiltration landscape of immune cells was constructed by ImmuCellAI, and the different abundance of 24 kinds of immune cells between HCC tissues and non-tumor tissues in the TCGA and GEO cohort was analyzed by Wilcoxon test. As shown in Figures 2A,B, majority of immune cells altered significantly in HCC, such as dendritic cells (DCs), macrophage, monocyte, CD4 T cell, natural killer cell, etc. Specific FDR testing results of each immune cell between two groups in both cohorts are provided in Supplementary Table S1. Then we perform the Kaplan–Meier analysis in order to find the immune cell with prognosis value. There are eight immune cells and one immune cell with prognosis value in TCGA and GEO cohort, respectively. After intersection with the differently infiltrated immune cells and the immune cells with prognosis value in both cohorts, we discovered that dendritic cells were the unique immune cells (Figure 2C). The Kaplan–Meier analysis was conducted in above two cohorts, and a high level of DC infiltration was founded robustly associated with poor prognosis (Figures 2D,E). Therefore, DCs were identified as the pivotal immune cells.

### Construction of Gene Co-expression Network

**FIGURE 3:**
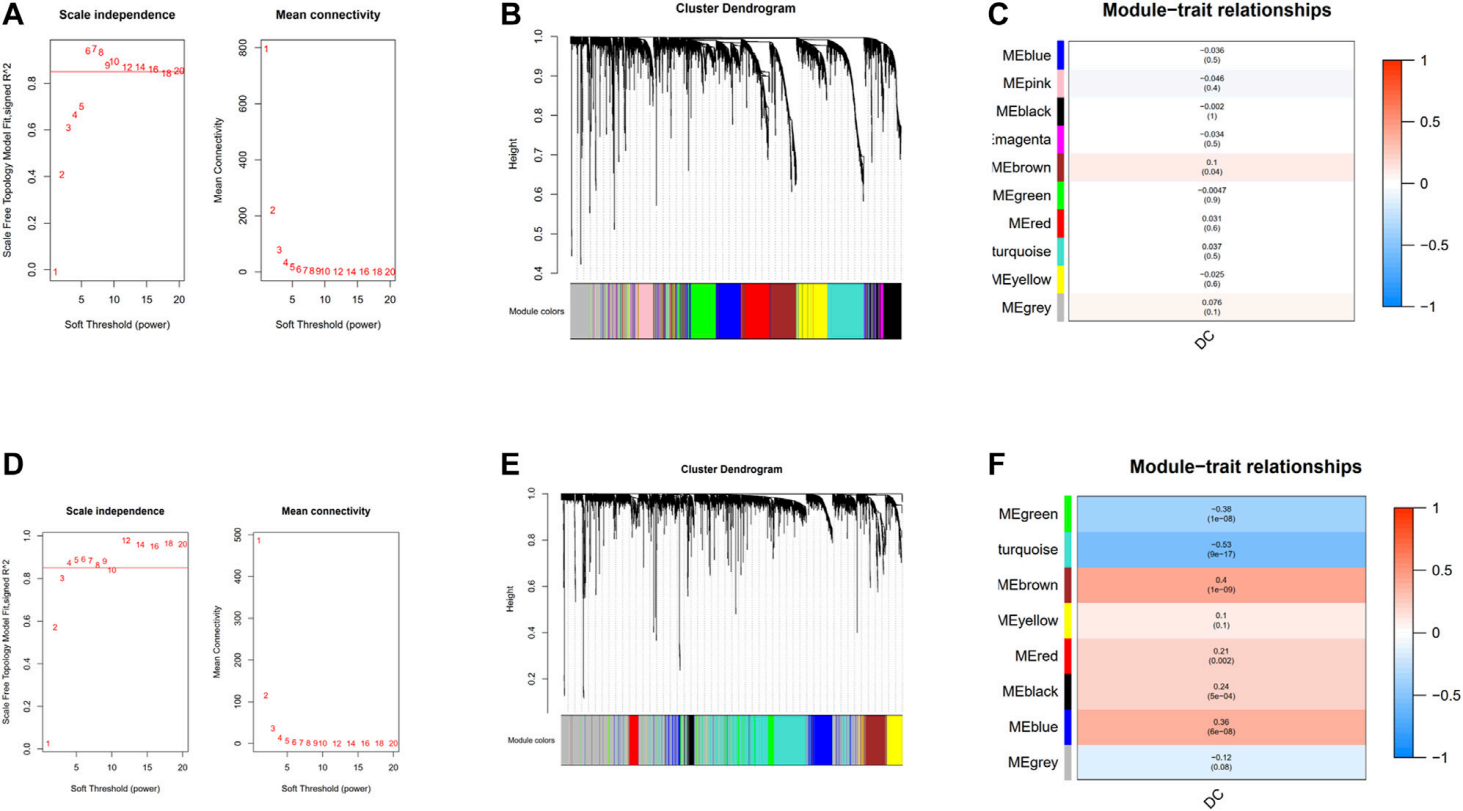
Construction of weighted gene co-expression network. **(A)** Analysis of the scale-free network coefficient R-squared for the soft threshold and the mean connectivity for the soft threshold in the TCGA cohort.**(B)** A cluster dendrogram of 10 network modules in the TCGA cohort. **(C)** Heat map of correlation between module eigengenes and DCs in the TCGA cohort. **(D)** Analysis of the scale-free network coefficient R-squared for the soft threshold and the mean connectivity for the soft threshold in the GEO cohort. **(E)** A cluster dendrogram of eight network modules in the GEO cohort. **(F)** Heat map of correlation between module eigengenes and DCs in the GEO cohort.

18,545 genes in TCGA and 13,423 genes in GEO with the most significant expression variance (top 25%) were extracted for subsequent WGCNA. In the TCGA cohort (Figure 3A), in order to ensure a scale-free network, the soft-thresholding power parameter was determined by the lowest power fit scale free index over 0.85, namely, *β* = 6 (scale-free R2 = 0.85). As for GEO cohort, *β* = 4 (scale-free R2 = 0.85) was the lowest power fit scale-free index over 0.85 (Figure 3D). Eventually, genes with similar expression patterns were grouped into 10 and 8 co-expression modules with different colors in TCGA and GEO cohort, respectively, *via* average linkage clustering (Figures 3B,E). In the TCGA cohort (Figure 3C), there was only one module (brown: r = 0.1, *p* = 0.04) significantly positively correlated with the abundance of DCs. In GEO cohort (Figure 3F), brown module (r = 0.4, p = 1e–09), red module (r = 0.21, *p* = 0.002), black module (r = 0.24, p = 5e−04), and blue module (r = 0.36, p = 6e−06) were the four modules that had significantly positive correlation with the abundance of DC cell.

### Identification of Hub Modules

**FIGURE 4:**
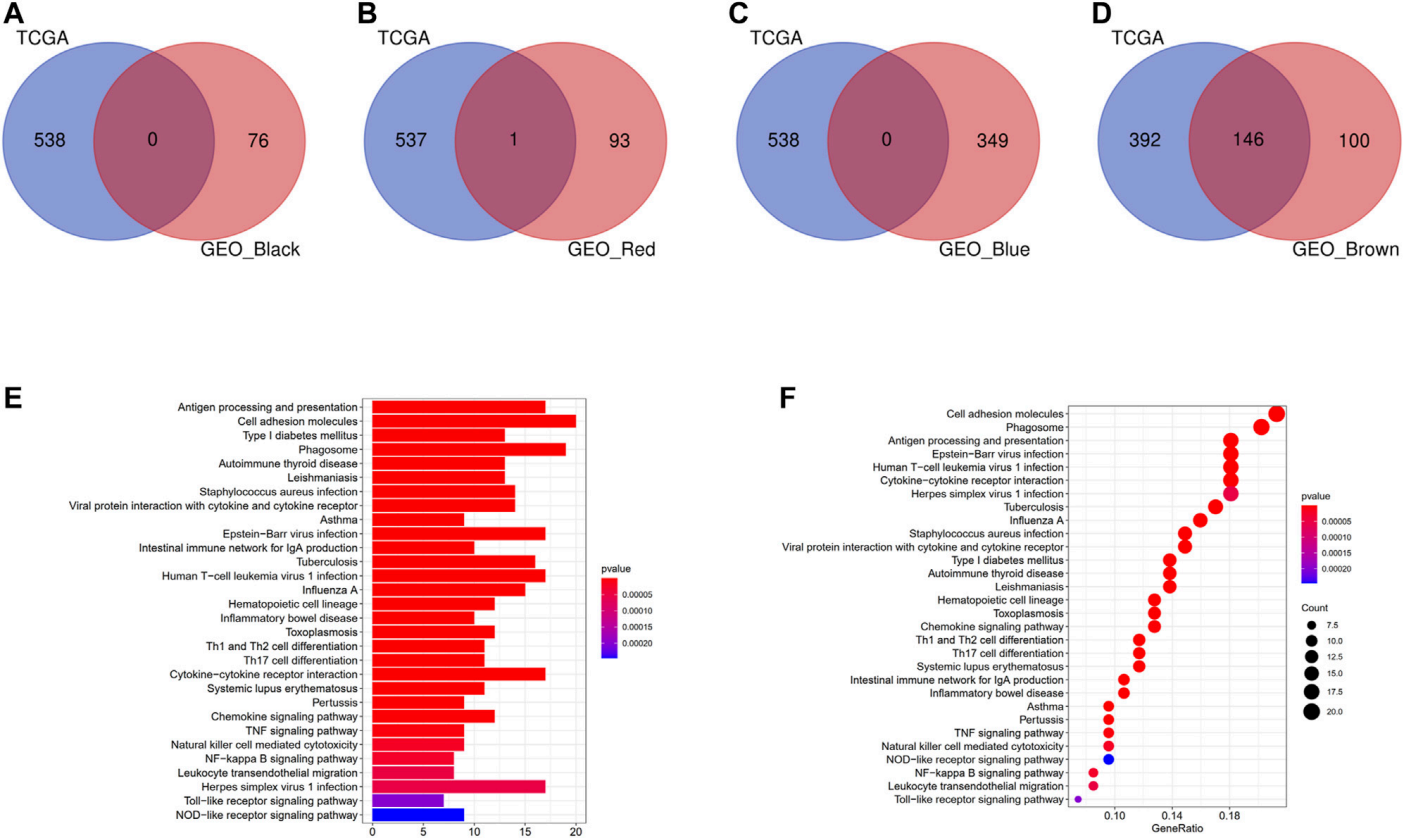
Identification of hub modules correlated with DCs infiltration. **(A)** Intersection of the brown module (TCGA) and black module (GSE14520). **(B)** Intersection of the brown module (TCGA) and red module (GSE14520). **(C)** Intersection of the brown module (TCGA) and blue module (GSE14520). **(D)** Intersection of the brown module (TCGA) and brown module (GSE14520). **(E)** Barplot of KEGG pathway enrichment analysis in the 146 overlapping genes. **(F)** Bubble plot of KEGG pathway enrichment analysis in the 146 overlapping genes.

Intersection of the modules with consistent correlation trend across TCGA and GEO cohorts was selected in the aim of identifying hub modules correlated with DC abundance [positive: brown module (TCGA) ∩ brown module (GSE14520), brown module (TCGA) ∩ red module (GSE14520), brown module (TCGA) ∩ black module (GSE14520), and brown module (TCGA) ∩ blue module (GSE14520)]. As shown in Figures 4A–D, there were 146 overlapping genes across brown module (TCGA) and brown module (GSE14520), while almost no overlapping gene in other intersections. KEGG pathway enrichment analysis indicated that the 146 overlapping genes were mainly enriched in immune-related pathways (Figures 4E,F). Therefore, brown modules in TCGA cohort and brown module in GEO cohort were regarded as hub modules correlated with DC infiltration, and their overlapping genes were subjected for the construction of prognostic model.

### Identification of Prognostic DC-Related DEGs in the TCGA Cohort

**FIGURE 5:**
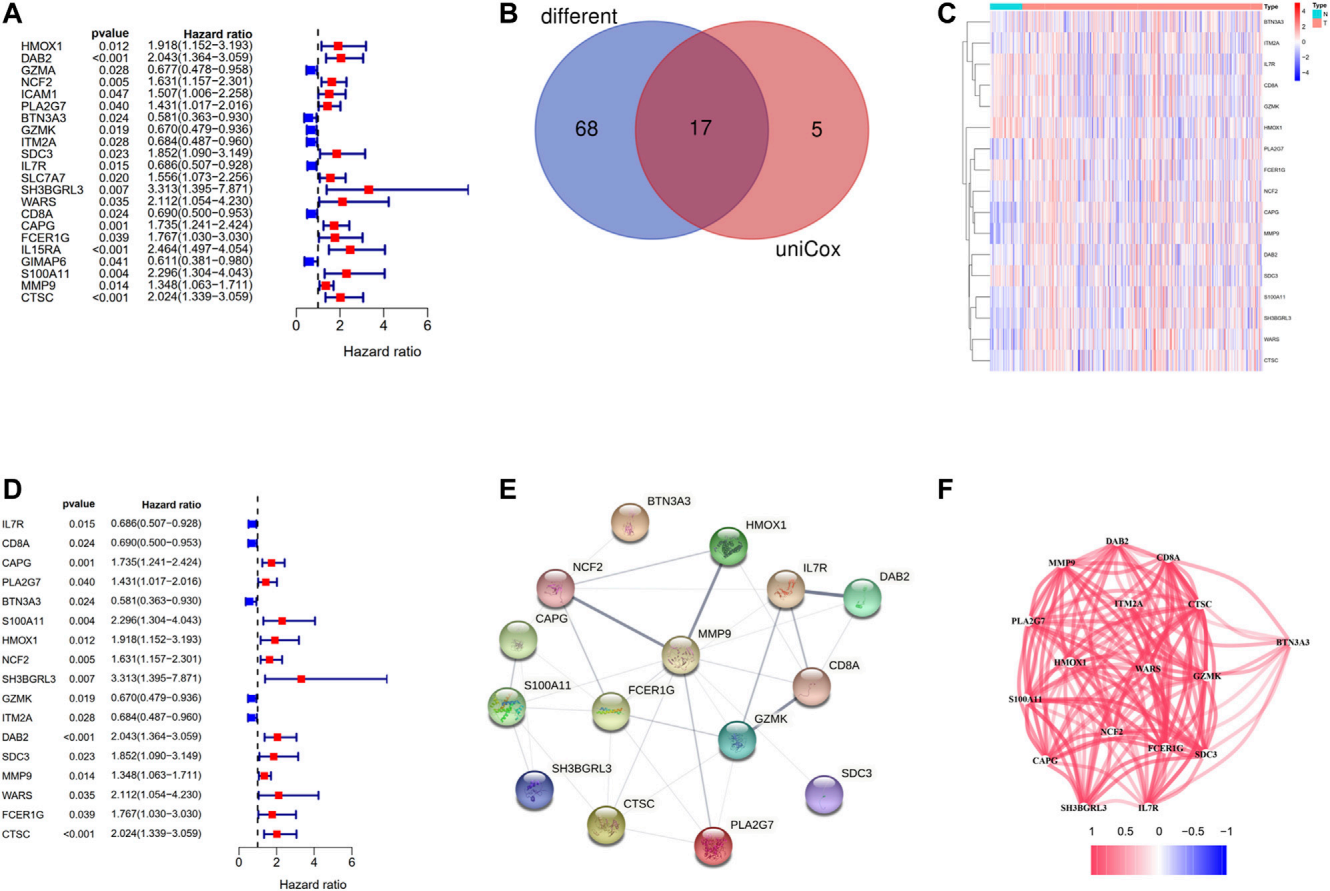
Identification of the candidate DC-related genes in the TCGA cohort. **(A)** The results of the univariate Cox regression analysis of 146 overlapping genes. **(B)** Venn diagram to identify differentially expressed genes between tumor and adjacent normal tissue that were correlated with OS. **(C)** The heat map of 17 prognostic DC-related DEGs. **(D)** The results of the univariate Cox regression analysis between gene expression and OS. **(E)** The PPI network of 17 prognostic DC-related DEGs downloaded from the STRING database. **(F)** The correlation network of 17 prognostic DC-related DEGs.

Among the 146 overlapping genes, 22 genes which were correlated with OS were obtained based on univariate Cox regression analysis (Figure 5A). Over half of the DC-related genes (85/146, 58.219%) were differentially expressed between HCC tissues and normal adjacent tissues, and 17 of them were associated with OS according to the univariate Cox regression analysis (Figure 5B). Among the 17 prognostic DC–related DEGs, 11 were upregulated,, while 6 were downregulated in tumor tissue, which was visualized using a heat map (Figure 5C). According to the univariate Cox regression analysis, all of the 17 genes were significantly associated with the OS of HCC patients, of which 12 indicated poor OS with elevating expression (HR > 1) and five suggested better OS with decreasing expression (HR < 1) (Figure 5D). We discovered that IL7R, HMOX1, NCF2, and DAB2 were the hub genes among these genes through the PPI analysis (Figure 5E). Furthermore, we calculated the correlation of expression level between these genes and found that all the involved genes were positively correlated (Figure 5F).

### Construction of a Prognostic Model in the TCGA Cohort

**FIGURE 6:**
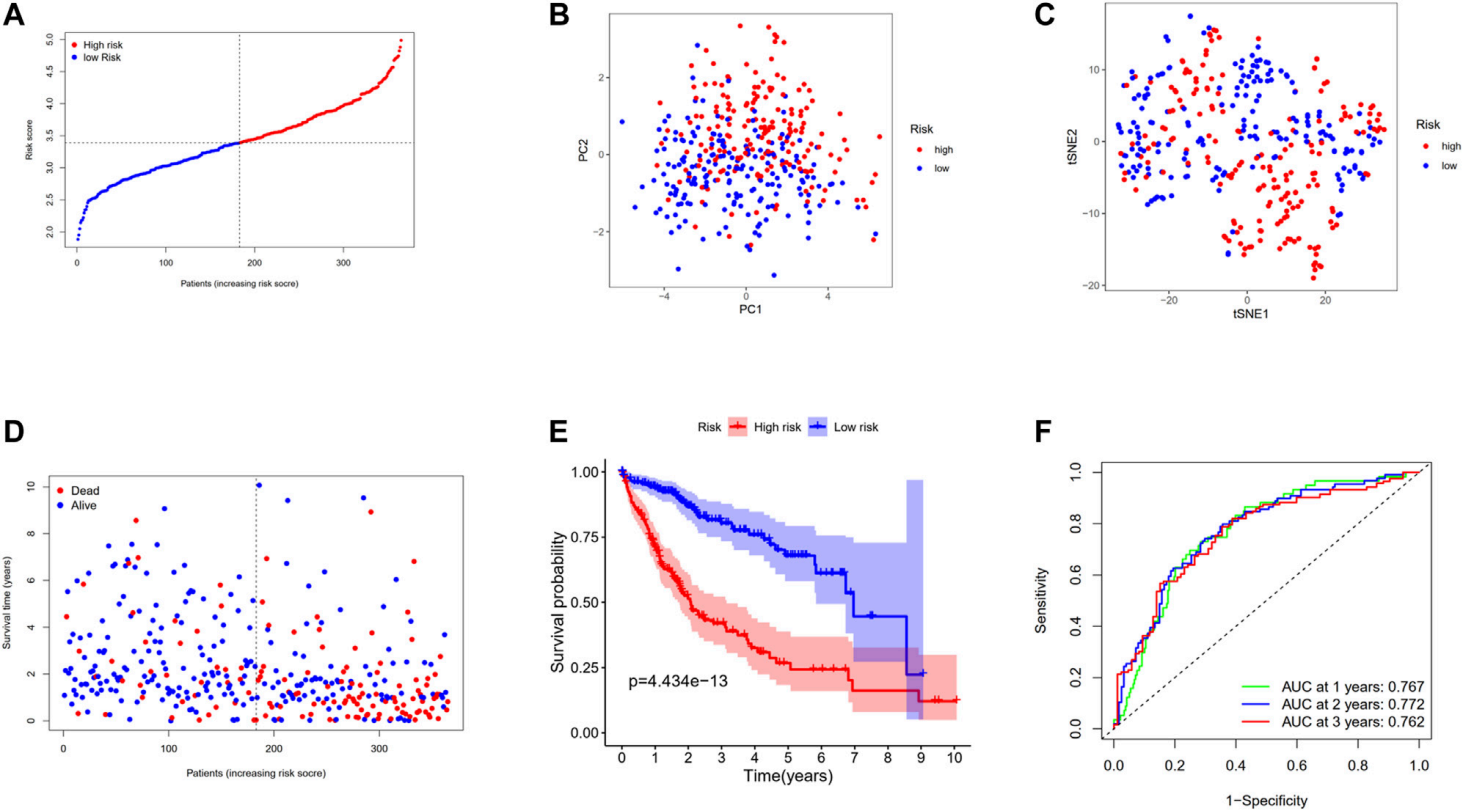
Prognostic analysis of the 12-gene signature model in the TCGA cohort. **(A)** The distribution and median value of the risk scores in the TCGA cohort. **(B)** PCA plot of the TCGA cohort. **(C)** t-SNE analysis of the TCGA cohort. **(D)** The distributions of OS status, OS, and risk score in the TCGA cohort. **(E)** Kaplan–Meier curves for the OS of patients in the high-risk group and low-risk group in the TCGA cohort. **(F)** AUC of time-dependent ROC curves verified the prognostic performance of the risk score in the TCGA cohort.

Through LASSO Cox regression analysis, the genes most contributing to the OS of HCC patients were screened out among the 17 genes mentioned above. According to the optimal value of λ, 12 predictors were finally identified. Then the DC-related prognostic model was constructed based on the following formula: risk score = *e* ^(−0.497 * expression level of IL7R+−0.692 * expression level of CD8A+ 0.232 * expression level of CAPG+0.186 * expression level of PLA2G7+−0.252 * expression level of BTN3A3+0.231 * expression level of HMOX1+0.031 * expression level of NCF2+0.246 * expression level of DAB2+0.234 * expression level of SDC3+0.028 * expression level of MMP9+0.909 * expression level of WARS+0.443 * expression level of CTSC)^. According to the median cutoff value, the patients in the TCGA primary cohort were stratified into a high-risk group (*n* = 182) or a low-risk group (*n* = 183) (Figure 6A). PCA and t-SNE analysis revealed that the patients in different risk groups were divided into two directions (Figures 6B,C). As presented in Figure 6D, high-risk patients are more likely to die earlier than low-risk patients. The Kaplan–Meier survival analysis also confirmed that the high-risk group had a significantly poor prognosis (Figure 6E, *p* < 0.001). The time-dependent ROC curves were utilized to make an evaluation of the performance of the gene signature for predicting OS and the area under the curve (AUC) achieved 0.767 at 1 year, 0.772 at 2 years, and 0.762 at 3 years (Figure 6F).

### Validation of the Prognostic Model in the GEO Cohort

**FIGURE 7:**
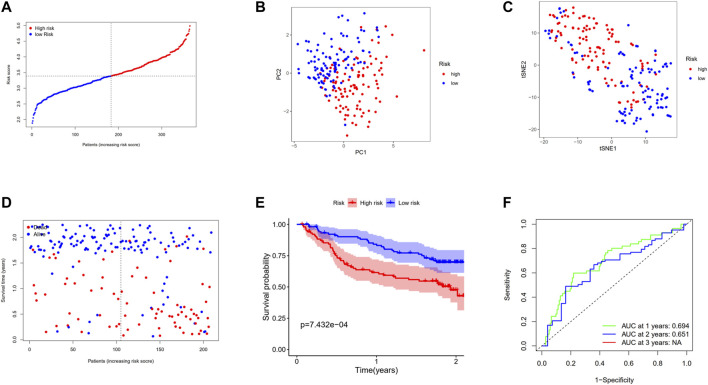
Validation of the 12-gene signature in the GSE14520 cohort. **(A)** The distribution and median value of the risk scores in the GSE14520 cohort. **(B)** PCA plot of the GSE14520 cohort. **(C)** *t*-SNE analysis of the GSE14520 cohort. **(D)** The distributions of OS status, OS, and risk score. **(E)** Kaplan–Meier curves for the OS of patients in the high-risk group and low-risk group. **(F)** AUC of time-dependent ROC curves in the GSE14520 cohort.

In the aim of testing the robustness of the model constructed by the TCGA cohort, we assessed the risk score of each patient in the GEO cohort with the aforementioned prognostic model. On the basis of the median value, the patients from the GEO cohort were divided into high-risk (*n* = 104) or low-risk groups (*n* = 105) (Figure 7A). The patients in the two subgroups were successfully separated confirmed by PCA and t-SNE analysis (Figures 7B,C). Similarly, patients in the high-risk group tended to suffer an earlier death (Figure 7D) and have a significantly shorter survival time than the low-risk group (Figure 7E, *p* < 0.001). In addition, the AUC of ROC analysis of the model at 1 and 2 years were 0.694 and 0.651, respectively (Figure 7F).

### Independent Prognostic Value of the Risk Score

**FIGURE 8:**
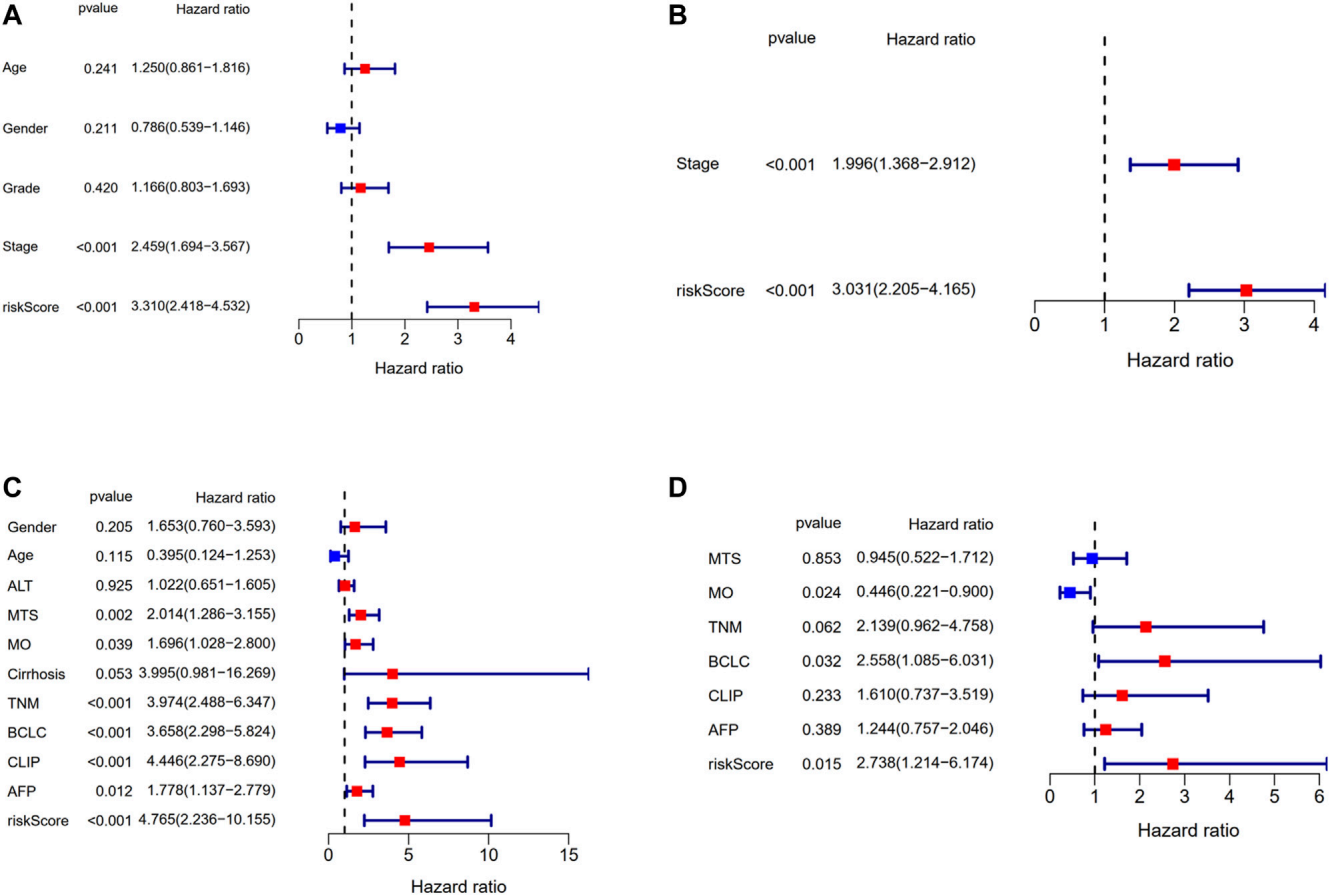
Results of the univariate and multivariate Cox regression analyses regarding OS in the TCGA derivation cohort and the GSE14520 validation cohort. **(A)** Univariate Cox regression analyses in the TCGA derivation cohort. **(B)** Multivariate Cox regression analyses in the TCGA derivation cohort. **(C)** Univariate Cox regression analyses in the GSE14520 validation cohort. **(D)** Multivariate Cox regression analyses in the GSE14520 validation cohort.

Univariate and multivariate Cox regression analyses were applied to assess whether the risk score was an independent prognostic factor for OS. The risk score had significant relationship with OS both in the TCGA cohort (HR = 3.310, 95% CI = 2.418–4.532, *p* < 0.001, Figure 8A) and the GEO cohort (HR = 4.765, 95% CI = 2.236–10.155, *p* < 0.001, Figure 8C) according to univariate Cox regression analysis. As for the multivariate Cox regression analysis where confounding factors were corrected, it indicated similarly that the risk score could serve as an independent predictor for OS (TCGA cohort: HR = 3.031, 95% CI = 2.205–4.165, *p* < 0.001; GEO cohort: HR = 2.738, 95% CI = 1.214–6.174, *p* = 0.015; Figures 8B,D).

### Functional Analyses in the TCGA and the GEO Cohorts

**FIGURE 9:**
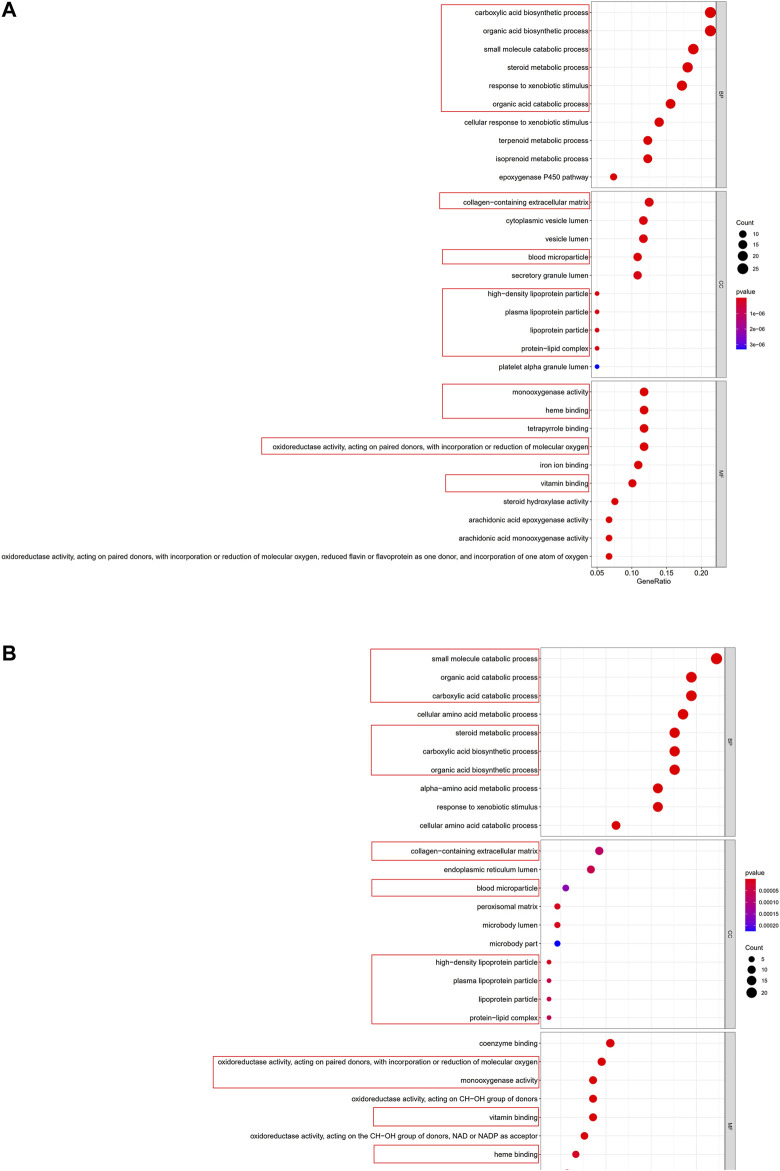
Representative results of GO analyses. **(A)** GO enrichment in the TCGA cohort. **(B)** GO enrichment in the GSE14520 cohort.

**FIGURE 10:**
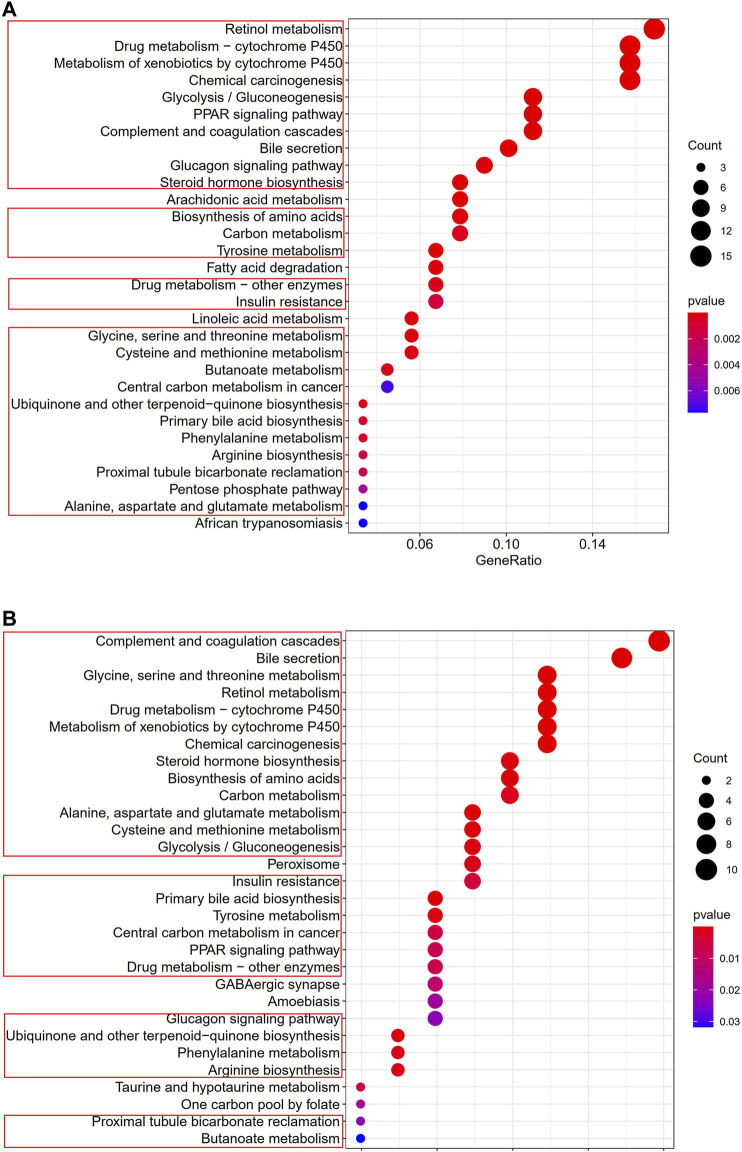
Representative results of KEGG analyses **(A)** KEGG pathways in the TCGA cohort. **(B)** KEGG pathways in the GSE14520 cohort.

To clarify the biological functions and pathways correlated with the risk score, the enrichment analysis of GO enrichment and KEGG pathway was implemented based on the DEGs between the high-risk and low-risk groups in TCGA and GEO cohorts. According to GO enrichment analysis, the DEGs between risk groups from the TCGA and GEO cohorts were mainly enriched in metabolic process (Figures 9A,B). KEGG pathway analysis also confirmed that the risk score was associated with various kinds of metabolism pathways (*P*. adjust <0.05, Figures 10A,B). The overlapped pathways were marked by red rim, and there were 25 overlapped pathways among the 30 KEGG pathways after we compared the functional analyses performed in both cohorts.

## Discussion

Our results revealed that high level of DC infiltration was associated with poor prognosis in patients with HCC, and a 12-gene risk-scoring model that was constructed based on the DC-related DEGs performed well in predicting OS in GEO cohort and TCGA cohort.

In this study, we first analyzed the different abundance of immune cells in HCC samples from GEO dataset and TCGA dataset, and then run the survival analysis of the differential immune cells. The results showed that the differential expression of DCs had a significant effect on OS in patients with HCC. The high expression of DCs indicates that the prognosis of patients with liver cancer is worse. Patients with high DC expression show worse prognosis than patients with low DC expression in HCC. DCs are regarded as crucial regulators of T-cell responses and involved in pathology *via* activating T cells and B cells (Hancock and et al., 2014; van Uden et al., 2019). Plasmacytoid dendritic cells (pDCs) have been documented in multiple primary and metastatic human cancers (Vermi et al., 2011). Infiltration by pDC of breast tumor correlates with dissemination and relapse, suggesting pDC contributes to the progression of breast cancer (Treilleux et al., 2004). On the contrary, depletion of pDC inhibits progression and bone metastasis of the breast cancer (Sawant et al., 2012). The administration of DCs has been applied to treat certain human neoplasms, such as melanoma and breast cancer (Luo et al., 2014). Zhou et al. (2019) revealed that intratumoral infiltration by pDCs has a predictive role for poor prognosis in patients with HCC.

In this study, the genes that co-expressed with DCs in GEO dataset and TCGA dataset were constructed by WGCNA. We found that the genes of black module, blue module, red module, and brown module in the GEO dataset were significantly correlated with DCs, and the genes of brown module in TCGA dataset were significantly correlated with DC. When the four significant DC-correlated modules of GEO dataset and the significant DC-correlated module of TCGA dataset were intersected, respectively, 146 genes were extracted as the fundamental genes that co-expressed with DCs, and these genes were analyzed by KEGG enrichment analysis. Since the genes co-expressed with DCs are positively correlated, and the high expression of DC predicts poor prognosis in patients with HCC, it is speculated that these 146 genes may also be closely related to the prognosis of patients with HCC. The enrichment pathways were mainly related to human T-cell leukemia virus-1 infection, phagosome, Th1 and Th2 cell differentiation, natural killer cell–mediated cytotoxicity, and leukocyte transendothelial migration, enhancing the reliability of these genes.

Through merging the univariable Cox regression analysis of 146 genes and differential gene expression analysis of 146 genes, we identified 17 genes retrieved as potential prognostic factors for constructing prognosis model. Afterward, LASSO Cox regression analysis was performed for constructing the risk-scoring model, and 12 genes (IL7R, CD8A, CAPG, PLA2G7, BTN3A3, HMOX1, NCF2, DAB2, SDC3, MMP9, WARS, and CTSC) with significant differential expression were selected. The risk-scoring model has favorable predictive validity in both GEO dataset and TCGA dataset. The 12-gene risk-scoring model may be a valuable prognostic factor for HCC patients. The high-risk group exhibits remarkably lower OS rate of HCC patients than the low-risk group. The AUC values of the risk-scoring model in GEO cohort and TCGA cohort showed benign performance in predicting short-term survival (1–2 years). Univariate and multivariate Cox analyses in the two cohorts together suggested that the 12-gene risk-scoring model performed a better prognostic value than other factors such as Barcelona Clinic Liver Cancer staging and TNM stages.

IL7R, whose expression was decreased in HCC, was considered to be a link to dedifferentiation of HCC and the top 50 predictor genes (Midorikawa et al., 2002). In our study, IL7R also downregulated in HCC samples and could be considered as a protective factor for HCC. CD8A was identified as one of the top 10 hub genes by bioinformatics analysis (Zhang and et al., 2017). CD8A showed significant positive correlation with most immune checkpoint–coding genes which closely related to the prognosis of HCC (Xu et al., 2020). We found the expression of CD8A was decreased in HCC samples and suggested better prognosis for HCC patients. CAPG, which could be detected in the cytoplasm of normal liver tissue and HCC specimens, might contribute to tumor motility and cancer-associated mortality and be regarded as a prognostic or diagnostic biomarker for metastatic HCC (Tsai et al., 2018). Although CAPG expression levels of normal tissues and tumor tissue without venous invasion were identical, its expression markedly upregulated in tumor tissue with vascular invasion compared to those without vascular invasion (Kimura et al., 2013). PLA2G7, as one of the secreted phospholipases A2, might provide potential HCC serological markers due to its strong upregulation in over half of HCC specimens (Smith et al., 2003). BTN3A3 has not been reported as its role of HCC. However, BTN3A3 was considered as a tumor suppressor gene, which could promote cellular apoptosis of nonsmall cell lung cancer (Jeon et al., 2016). Besides, high level of BTN3A3 expression was correlated with better disease-free survival (DFS) and OS of gastric cancer patients (Pan et al., 2019). HMOX1 is the inducible isoform of the rate-limiting enzyme in heme degradation (Gueron et al., 2009). HMOX1 was involved in invasion and metastasis of multiple cancers. HMOX1 suppress breast cancer invasion through inhibiting the expression of matrixmetalloproteinase-9 (MMP9) (Lin et al., 2008). It was proved to be a prognostic factor for HCC patients with hepatectomy (Yeh et al., 2018). HMOX1 might inhibit the proliferation and metastasis of HCC by regulating the miR-30 days/miR-107 level (Zou et al., 2016). Hence, the downregulation of HMOX1 found in this study was fitted with the results of current researches. At present, research on the relationship between NCF2 and HCC was still absent. NCF2 potentially provided pathological diagnostics and prognostic value of cervix carcinogenesis (Lomnytska et al., 2011). Furthermore, upregulation of NCF2 could promote gastric cancer, angiogenesis, and metastasis (Zhang et al., 2018). Disabled homolog 2 (DAB2), as a member of the disable gene family, has been proven to function as a tumor suppressor that plays an crucial role in the occurrence and progression of various tumors (Albertsen et al., 1996), including colorectal cancer (Kleeff et al., 2002) and epithelial ovarian cancer (Mok et al., 1998). Besides, DAB2 is highly expressed in tumor-infiltrating tumor-associated macrophages (Marigo et al., 2020). DAB2 may attenuate the miR-106b promotion effect on HCC cell proliferation and migration. Downregulation of DAB2IP is associated with poor prognosis in HCC patients, which represents that DAB2IP is a considerable marker for progression of HCC (Zhang et al., 2012; Chen Y. et al., 2019). Until recently, SDC3, as one of the hypoxia-related gene, along with other 13 genes was found to be a potential prognostic biomarker for breast cancer (Wang et al., 2020). Zong et al. (2010) found that overexpression of SDC1 inhibits the proliferation of mesenchymal tumor cells. In this study, we revealed that the expression of SDC3 was downregulated in HCC patients. Matrix metalloproteinases (MMPs), an important proteolytic event in the invasion and migration of tumors, is associated with the degradation of the extracellular matrix (Bonnans et al., 2014). As one of fundamental member of MMPs family, MMP9 significantly contributed to the progression of multiple tumors in the context of overexpression (Yan et al., 2018; Dai et al., 2019; Zhou et al., 2019). Liu et al. (2020) identified that M2 macrophages promoted HCC cells invasion and metastasis through upregulating MMP9 expression, which suggested elevating MMP9 expression was correlated with immune related cells in TME. WARS, as an aminoacyl-tRNA synthetase and inhibitor of angiogenesis, encodes the human cytoplasmic tryptophanyl-tRNA synthetase (TrpRS) and participates in protein synthesis and RNA transcription as well as translation (Tsai et al., 2017). Low expression of TrpRS in tumor tissue was associated with worse outcomes in patients with colorectal cancer and pancreatic cancer (Ghanipour et al., 2009; Paley et al., 2011). Cathepsin B(CTSB) might be associated with the growth and metastasis of HCC as an oncogene and serve as a valuable prognostic marker for HCC patients (Ruan and et al., 2016; Zhang et al., 2020). Evidence supported that overexpression of CTSB predicted poor prognosis of numerous cancer patients, including HCC patients (Ruan and et al., 2016).

Taken together, the 12-gene risk-scoring model may be a valuable prognostic factor for HCC patients. Among the 12-gene risk-scoring model, SDC3, NCF2, BTN3A3, and WARS have never been reported as the prognostic factor for HCC. Although the prognostic model associated with the 12 genes has not been reported previously and they could be considered as a valuable prognostic method for HCC, this study has several limitations. First, the raw data on HCC that we downloaded from GEO dataset was limited and incomplete. Second, the long-term survival predictive value of the 12-gene risk-scoring model was obscured.

## Conclusion

Our finding revealed that the 12-gene risk-scoring model could serve as a potential prognostic prediction for HCC. SDC3, NCF2, BTN3A3, and WARS were noticed as a novel prognostic factor for HCC.

## Data Availability

The datasets presented in this study can be found in online repositories. The names of the repository/repositories and accession numbers can be found below: https://www.ncbi.nlm.nih.gov/geo/, GSE14520 https://xenabrowser.net/datapages/, TCGA-LIHC. htseq_fpkm.tsv.

## Glossary

HCC: hepatocellular carcinoma
HBV: hepatitis B virus
TME: tumor microenvironment
CTLs: cytotoxic T lymphocytes
LASSO: absolute shrinkage and selection operator
ROC: receiver operating characteristic
PCA: principal component analysis
t-SNE: t-Distributed Stochastic Neighbor Embedding
OS: Overall survival
GEO: Gene Expression Omnibus
TCGA: The Cancer Genome Atlas
ImmuCellAI: Immune Cell Abundance Identifier
MEs: module eigengenes
FDR: false discovery rate: false discovery rate
BH: Benjamini & Hochberg
PPI: protein–protein interaction network
WGCNA: Weighted gene co-expression network analysis
KEGG: Kyoto Encyclopedia of Genes and Genomes
FDR: false discovery rate: false discovery rate
DEGs: differentially expressed genes
BP: biological process
CC: cellular component
MF: molecular function
DCs: dendritic cells
pDCs: plasmacytoid dendritic cells
AUC: area under the curve
DFS: disease free survival
HR: hazard ratio
CI: confidence interval
IL7R: interleukin 7 receptor
CD8A: CD8a molecule
CAPG: gelsolin-like capping actin protein
PLA2G7: phospholipase A2 group VII
BTN3A3: butyrophilin subfamily 3 member A
HMOX1: heme oxygenase 1
NCF2: neutrophil cytosolic factor 2
DAB2: disabled homolog 2
DAB2IP: DOC-2/DAB2 interactive protein gene
SDC3: syndecan 3MMPs:Matrix metalloproteinases
MMP9: matrixmetalloproteinase-9
TrpRS: tryptophanyl-tRNA synthetase
CTSC: cathepsin C.
